# Current and New Perspectives in the Diagnosis of Blastomycosis and Histoplasmosis

**DOI:** 10.3390/jof7010012

**Published:** 2020-12-29

**Authors:** Kathleen A. Linder, Carol A. Kauffman

**Affiliations:** Infectious Diseases Section, Veterans Affairs Ann Arbor Healthcare System, University of Michigan Medical School, Ann Arbor, MI 48105, USA; ckauff@med.umich.edu

**Keywords:** blastomycosis, *Blastomyces dermatitidis*, histoplasmosis, *Histoplasma capsulatum*, antigen detection

## Abstract

The diagnosis of blastomycosis and histoplasmosis can be difficult for clinicians who rarely see infections caused by these environmentally restricted dimorphic fungi. Historically, the diagnosis of blastomycosis has been established by culture and sometimes by histopathologic identification. Currently, antigen detection in urine and serum has been shown to aid in the rapid diagnosis of blastomycosis, and newer antibody assays are likely to contribute to our diagnostic capability in the near future. The gold standard for the diagnosis of histoplasmosis has been culture of the organism from involved tissues, aided in some patients by histopathological verification of the typical yeast forms in tissues. Antigen detection has contributed greatly to the ability of clinicians to rapidly establish the diagnosis of histoplasmosis, especially in severely ill and immunocompromised patients, and antibody testing for *Histoplasma capsulatum* provides important adjunctive diagnostic capability for several forms of both acute and chronic histoplasmosis. For both of these endemic mycoses, novel molecular tests are under active investigation, but remain available in only a few reference laboratories. In this review, we provide a synopsis of diagnostic test options that aid in establishing whether a patient has blastomycosis or histoplasmosis.

## 1. Blastomycosis

Recent advances in taxonomy have established that there are several species in the genus *Blastomyces*. *Blastomyces dermatitidis* and *Blastomyces gilchristii* are closely related organisms that cause indistinguishable clinical disease and have similar epidemiological niches [1]. The differences between these two species can be shown only by analyzing their genetic sequences; phenotypically, they are identical, and clinical laboratories cannot differentiate between them. The geographic distribution of these *Blastomyces* species includes areas around the Great Lakes, the Mississippi and Ohio River Valleys, and the St. Lawrence seaway. *B. gilchristii* appears to be the predominant species in several localized regions in northwestern Ontario and northern Wisconsin, where large outbreaks of blastomycosis have occurred [2,3]. For clinical and diagnostic purposes, these can be viewed as the same organism, and we will refer to them both as *B. dermatitidis* throughout this review.

There now are several other species in the genus *Blastomyces;* these organisms differ from *B. dermatitidis* in both clinical and microbiological aspects. *Blastomyces helicus* was initially named *Emmonsia helica* when it was first described in 2015 [4], and a few years later it was transferred to the genus *Blastomyces* [5]. This organism has been reported to cause disease mostly in immunocompromised patients, including those who have HIV, a hematological malignancy, a solid organ transplant, or are taking immunosuppressive agents; the clinical characteristics of infection differ from those typically seen with blastomycosis [6]. The epidemiology of this species also differs from that of *B. dermatitidis* in that cases have been reported from the Western United States and Canada, areas outside the classic endemic region for *B. dermatitidis*. *B. helicus* differs morphologically from *B. dermatitidis* in both the mold and yeast phases. The *B. helicus* yeast forms are smaller than those of *B. dermatitidis*, and cells can have multiple buds. In the mold phase, conidia are absent and hyphae can form characteristic spirals under some conditions [6].

*Blastomyces percursus* is another newly described dimorphic fungus placed in the *Blastomyces* genus [7]. The epidemiology of this species differs from that of *B. dermatitidis* in that infection has been reported to date only in a few patients living in Africa and the Middle East. The mold phase differs from that of *B*. *dermatitidis*, but the yeast phase of these two species appears similar in tissues [5,7].

The latest addition to the *Blastomyces* genus was added in 2018. The environmental mold *Emmonsia parva* has been reclassified as *Blastomyces parvus* [8]. This organism is dimorphic, as are all *Blastomyces* species; it does not form yeast-like organisms in tissues, but rather large, swollen, structures called adiaspores [9]. This organism, although genetically related, does not cause the typical clinical picture seen with blastomycosis, but instead causes adiaspiromycosis, a rare granulomatous pulmonary infection [10].

Because these organisms are so uncommon, the atypical *Blastomyces* species should only rarely be entertained as a diagnostic possibility, and even then only when the patient has not been in an area known to harbor *B. dermatitidis* or when the histopathology or culture information are not typical for *B. dermatitidis.* The discussion on diagnosis that follows will focus only on *B. dermatitidis.*

## 2. Diagnosis of Blastomycosis

The diagnosis of blastomycosis relies primarily on the standard methods of culture and histopathology, combined with a history of possible exposure to this environmental fungus [11,12,13]. Antigen testing has proved useful for diagnosis, but antibody assays have been notoriously insensitive and nonspecific. Molecular techniques have had some use, but remain non-standardized.

### 2.1. Culture

The definitive diagnosis of blastomycosis remains the isolation of *B. dermatitidis* in culture. If disease is primarily pulmonary, sputum can provide an adequate sample for culture for some patients, but bronchoalveolar lavage (BAL) fluid is more likely to yield the organism. When skin lesions are present, culture of biopsy material will often yield the organism. Dissemination to sites other than the skin is less common, but in some patients, material for culture can be obtained from biopsy of prostate, bone, or other involved tissue or from the aspiration of synovial fluid. Patients who have central nervous system symptoms should have cerebrospinal fluid (CSF) cultured, but the yield is low [14].

Material from any of the sources noted above should be placed on Sabouraud’s dextrose agar, kept at room temperature for 4 to 6 weeks, and assessed for growth of a white or tan mold. Once growth occurs, a commercially available chemiluminescent labeled DNA probe (AccuProbe, Hologic Inc., San Diego, CA, USA) that hybridizes to rRNA of *B. dermatitidis* can quickly confirm the identity of the organism. Not surprisingly, *B. gilchristii* tests positive with this probe, and also *B. helices* [6]. Additionally, *Emergomyces canadensis* and *Paracoccidioides brasiliensis,* two other dimorphic environmental fungi, can test positive with this probe, but have different morphologic appearances than *Blastomyces* [15,16]. Rarely encountered fungi, such as *Gymnascella hyalinospora* and *Spiromastigoides asexualis,* also can give false positive tests with the DNA probe [17,18].

### 2.2. Histopathology and Cytology

The yeast or tissue form of *B. dermatitidis* is quite distinctive, allowing a diagnosis of proven blastomycosis before the culture has yielded growth of the organism under the most recent European Organization for Research and Treatment of Cancer and the Mycoses Study Group Education and Research Consortium (EORTC/MSGERC) consensus guidelines [19]. This approach allows earlier diagnosis and definitive treatment, which is especially important in severely ill patients. The distinctive yeast form is thick-walled, relatively large (typically 8–15 µm), and has a single budding daughter cell that is attached to the mother cell by a broad base. Because of the size, these structures can sometimes be seen on hematoxylin and eosin staining, but definite delineation of the organism always should be sought by staining tissues with a methenamine silver stain or periodic acid-Schiff (PAS) stain. When fluid is obtained for diagnostic purposes, *B. dermatitidis* is best seen with a calcofluor white stain or in cytological preparations stained with Papanicolaou stain [12]. In general, the tissue response in patients with blastomycosis is pyogranulomatous; if skin is involved, pseudoepitheliomatous hyperplasia is characteristic.

### 2.3. Antibody Testing

Standard immunodiffusion (ID) and complement fixation (CF) assays that are useful for the diagnosis of histoplasmosis have not proved to be sensitive or specific enough to aid in the diagnosis of blastomycosis [20,21]. Further refinements that focused on measuring the antibody response to the WI-1 (BAD-1) adhesion antigen appeared to be more promising [22]. An enzyme immunoassay (EIA) targeting the BAD-1 surface protein offered higher sensitivity and specificity than prior assays [23]. Initial reports with this new assay noted a sensitivity of 88%, and specificity was 99%; however, this was a comparison with serum from patients who either did not have a fungal infection or were healthy controls. When serum samples from patients with histoplasmosis were tested, positive results were found in 3 of 50 samples. Confirmation of sensitivity and specificity await larger studies using this antibody assay.

### 2.4. Antigen Detection

An enzyme immunoassay that detects galactomannans in the cell wall of *B. dermatitidis* (MiraVista Diagnostics, Indianapolis, IN, USA) is available in the United States [24,25]. This assay can be used to test urine, serum, BAL fluid, and CSF [26,27,28]. Most data on sensitivity and specificity are reported for urine, for which the sensitivity has varied from 76% to 90% in different reports [24,27,28,29]. The test is less sensitive in serum, varying from 56% to 82% [25,27,28]; pretreatment with EDTA and heating increases the sensitivity [25]. Sensitivity for antigen detection in BAL fluid and CSF are not known, but have been reported to aid in diagnosis in individual cases [26].

The assay appears highly specific for endemic mycoses, but because many endemic fungi share galactomannan cell wall antigens, the specificity for individual genera is very low. For example, cross-reactivity between the cell wall antigens of *H. capsulatum* and *B. dermatitidis* is reported to be 93–96% using this assay [24,25]. Although several patients with blastomycosis have been reported to have false positive antigen tests for *Aspergillus* galactomannan, no patients with aspergillosis have been reported to have a positive antigen assay for *B. dermatitidis* [30,31].

Several authors have suggested that following antigen levels in urine can be useful for monitoring the resolution or progression of infection [28,32,33]. In dogs treated for blastomycosis, a decrease in both urine and serum antigen levels has been associated with clinical improvement [34].

### 2.5. Nucleic Acid Tests

Polymerase chain reaction (PCR) has been used infrequently as an aid in the diagnosis of blastomycosis. PCR is very specific and can confirm the diagnosis of blastomycosis, particularly in cases where blastomycosis and histoplasmosis are both possible. However, PCR assays are not standardized or generally available. A few reference laboratories offer PCR testing for *B. dermatitidis* using assays developed by those institutions [35]. An assay that targets the *BAD*1 adhesin gene has proved to have high sensitivity and specificity when used to test environmental samples, paraffin-embedded tissues from dogs with blastomycosis, and a small number of human tissue samples [36,37,38]. It is likely that molecular techniques will eventually become more standardized and may become useful rapid assays for blastomycosis.

## 3. Histoplasmosis

Histoplasmosis is caused by fungi of the genus *Histoplasma*; the most common is *Histoplasma capsulatum* var. *capsulatum* (hereafter referred to as *H. capsulatum*). This fungus can be found worldwide, but the highest rates of exposure are found in North and Latin America [39]. In North America, the highest exposure rates occur in the Ohio and Mississippi river valleys, as shown by skin testing with histoplasmin [40,41]. In Latin America, areas with high exposure rates include southern Mexico, Central America, and northern South America. *Histoplasma capsulatum* var. *duboisii* has a much more limited geographic distribution, occurring mostly in Central and West Africa, where it coexists with *H. capsulatum* [42].

Most patients who are exposed to *H. capsulatum* do not develop disease. A small percentage develop acute pulmonary histoplasmosis, which manifests with fevers, malaise, dry cough, patchy pulmonary infiltrates, and lymphadenopathy [43]. Immunocompromised patients, including those with advanced HIV or following organ transplantation, often develop severe pulmonary infection or widespread disseminated infection [44,45].

## 4. Diagnosis of Histoplasmosis

### 4.1. Culture

The gold standard for the diagnosis of histoplasmosis is the recovery in culture of *H. capsulatum* from a clinical specimen. Growth of *Histoplasma* may take as long as 6 weeks. Initial growth appears as a white-tan colony at 25 °C on Sabouraud’s dextrose agar. A lactophenol cotton blue preparation will first demonstrate septate hyphae, then eventually microconidia (2–4 µm) and the characteristic tuberculate macroconidia (8–15 µm). Rarely, mold cultures produce only the smooth walled microconidia and some fail to sporulate. Incubation at 37 °C causes transformation from the mold phase to the yeast phase. However, transformation to the yeast phase is not needed for definitive identification because a commercially available DNA probe with a chemiluminescent label that binds to *H. capsulatum* rRNA can quickly identify the organism (AccuProbe, Hologic Inc., San Diego, CA, USA). False-positive tests are rare but have been reported with *Chrysosporium* spp. [46]. Use of matrix-assisted laser desorption ionization time of flight mass spectrometry (MALDI-ToF MS) technology for organism identification is being studied as an alternative to the use of a DNA probe; identification is possible but due to the infectivity of the organism, this technique may not be viable for clinical laboratories [47].

Although culture is the gold standard, overall sensitivity is low. The yield of *H. capsulatum* in different forms of histoplasmosis varies tremendously. For example, in mild to moderate pulmonary infection, cultures are often negative, and in central nervous system disease, cultures are rarely positive [43,48]. Additionally, commensal organisms frequently overgrow cultures from specimens taken from the respiratory tract [43].

*H. capsulatum* can be grown from blood, but only rarely when conventional blood culture systems are used and not within the time period of 5 days that blood culture bottles are typically incubated; the average time to growth of *H. capsulatum* in cultures of blood is between 12 to 15 days [49]. The use of a lysis-centrifugation system greatly improves detection from blood [49,50,51]. Hyphal forms and large bizarre yeast forms, rather than typical small oval yeasts, can be seen on smears made from blood cultures.

### 4.2. Histopathology and Cytology

On histopathology of a tissue specimen, *H. capsulatum* appears as a 2–4 µm narrow-based budding yeast when methenamine silver or PAS stains are used. It is poorly seen on Gram stain [52]. For bone marrow specimens or touch preparations, a Giemsa stain is very helpful to show the yeast forms. Similar to blastomycosis, the organism is distinctive enough to allow for a diagnosis of proven histoplasmosis by the EORTC/MSGERC consensus guidelines [19]. The organisms are typically intracellular, but have also been noted in extracellular spaces. The species name *capsulatum* is a misnomer; the original reports of the organism by Samuel Darling in 1906 describe a non-staining area around the organism, hence the name *H*. *capsulatum* [53]. *H. capsulatum* is non-encapsulated, and the “capsule” that was noted was an artifact of tissue processing. Granulomatous inflammation, often with necrosis, is typically seen, except when patients are markedly immunosuppressed, as occurs with advanced AIDS; non-necrotizing or incomplete granulomas may form in these cases when the inflammatory response is impaired. In endovascular infections, particularly endocarditis, atypical and aberrant yeast forms and hyphae are often seen in tissue [54].

### 4.3. Antibody Testing

Antibody testing for *H. capsulatum* is most helpful after the acute phase of disease, as antibodies can be detected 4–8 weeks after initial infection [55]. Serologic testing is most useful in immunocompetent hosts and is limited in patients who are unable to produce a reliable antibody response, such as those taking immunosuppressive drugs or following solid organ transplantation [44].

Standard antibody assays include both CF and ID. The CF assay tests for both yeast and mycelial (histoplasmin) antibodies [56]. A fourfold increase in either antibody is considered positive; a single value of ≥1:32 is suggestive of histoplasmosis but is not diagnostic [43]. The ID assay assesses the presence of H and M bands [43,51,56]. The H band is seen in <20% of cases, rarely without the M band, and is noted in more severe acute pulmonary or disseminated infection and in chronic forms of histoplasmosis, such as chronic cavitary pulmonary histoplasmosis. The M band is more commonly seen, appears earlier in the course of infection, and can persist for several years after the H band has disappeared and infection has resolved.

The CF assay, particularly the yeast phase, is slightly more sensitive than ID for the diagnosis of histoplasmosis; however, sensitivity for both the ID and the CF yeast phase tests has been noted to be as high as 90% in some studies [56]. The CF mycelial phase antibody is the most specific of the assays but has poorer sensitivity.

Early studies using a more cumbersome radioimmunoassay showed the potential diagnostic benefit of an assay that could measure IgG and IgM antibodies to *H. capsulatum* [57]. This led to the development of an easily performed EIA to detect IgM and IgG antibodies to *H. capsulatum* [58]. The sensitivity of this commercially available EIA assay (MiraVista Diagnostics, Indianapolis, IN, USA) is 77–96% and the specificity is 92%. The EIA test appears to be more sensitive than ID or CF when compared using samples from the same patient. This EIA assay is of special utility in patients with *Histoplasma* meningitis, in which antibodies to *Histoplasma* in the cerebrospinal fluid may be the only indication of disease [47]. Cross-reactivity has been noted in patients who have blastomycosis or coccidioidomycosis [58]. Several studies have noted that the combination of the EIA antibody assay and the antigen assay further improves diagnostic accuracy [47,58].

### 4.4. Antigen Detection

The development of antigen testing for histoplasmosis has revolutionized the diagnosis of histoplasmosis. Antigen testing was first developed in the late 1980s using a sandwich radioimmunoassay, which was reformulated into an EIA in 1989. A semi-quantitative second-generation test was developed in 2004 and followed in 2007 by the currently available third-generation, quantitative antigen assay (MiraVista Diagnostics) [59,60]. A second antigen assay using the lateral flow technique has been developed by IMMY Diagnostics (Norman, OK, USA). Testing by MiraVista requires submission of the sample to a central laboratory, but testing with the IMMY platform can be done by individual laboratories, which is useful in settings where processing and shipping samples is not feasible [61,62]. A direct comparison of the sensitivity and specificity of the two assays has not been performed. Rapid antigen testing is of critical importance in areas, such as Latin America, in which mortality rates from histoplasmosis are especially high in persons living with HIV/AIDS [45].

Antigen testing is most frequently done on urine or serum specimens and is of highest yield in patients with disseminated histoplasmosis; in this population, antigen is present in the urine in approximately 90% of patients and in the serum in approximately 80% of patients [60]. Antigen is less commonly detected in acute pulmonary histoplasmosis; in one report, antigen was positive in urine in only 65% of patients and in serum in 69% of patients [63]. Antigen testing is often negative in patients with chronic cavitary pulmonary histoplasmosis and is almost always negative in patients with local complications of histoplasmosis, such as mediastinal granuloma [43]. Antigen testing, particularly in urine, can be used to monitor response to therapy, but some patients who have been successfully treated will show persistence of low concentrations of urinary antigen for many months [60,64]. A rise in antigen levels can indicate disease relapse [65]. *Histoplasma* galactomannans are similar to antigens of other dimorphic fungi, including *B. dermatitidis*, *P. brasiliensis*, *Talaromyces marneffei*, and *Sporothrix* species, and cross-reactivity of these fungi with the antigen test is common [66,67]. This is most clinically relevant for *B. dermatitidis* infection, given overlapping areas of endemicity for these fungi. *Aspergillus* galactomannan testing also cross-reacts with *Histoplasma* galactomannans and can be positive in patients with histoplasmosis; patients with aspergillosis do not have false positive *Histoplasma* antigen [68].

Antigen testing can be performed on other samples, including BAL fluid and CSF [47,69]. It is highly specific and sensitive when CSF is tested in patients with *Histoplasma* meningitis, in which culture is often negative [47]. Test characteristics are similarly good when used on BAL fluid; in some instances, antigen can be detected in BAL fluid when culture is negative for *H. capsulatum* [69].

### 4.5. Nucleic Acid Tests

PCR tests have been developed for the detection of *H. capsulatum* but are not often used in clinical care. *H. capsulatum* rDNA, which is a commonly used target for PCR, is similar to 18-S rDNA of other fungi [70]. This has led to the use of nested PCR, in which multiple primer sets are used. Initial PCR is done for the 18-S rDNA, then PCR is done on that amplified product to detect a more specific product for *H. capsulatum*. Because amplification of an already amplified product occurs, there is an increased risk of contamination [70,71]. Early studies of nested PCR mechanisms testing for both *H. capsulatum* rDNA and a 100-kDa-like protein unique to *Histoplasma* (later named *Hcp100*) suggested excellent specificity, although they were limited by DNA extraction capabilities [70]. Subsequent studies have also been done using N-acetylated α-linked acidic dipeptidase (NAALAD) and the internal transcribed spacer (ITS) region [72,73,74]. Sensitivity appears to be highest with PCR of the ITS region in both patients with and without HIV infection; *Hcp100* and NAALAD are less reliable [73,75]. However, most studies on the use of PCR are small studies done primarily in patients with advanced HIV infection. These data may not be generalizable. PCR testing has not been well validated and is not available for widespread clinical use.

Traditional PCR techniques are not feasible in all settings, on account of their need for specialized equipment and trained operators, and techniques that replicate DNA sequences at a constant temperature may be preferred in resource-limited settings. Loop-mediated isothermal amplification (LAMP) is one such technique that can be completed in one tube and in less than an hour with high selectivity for target DNA [76]. This assay can be modified with the addition of fluorescent markers or dyes to allow easy interpretation [77,78]. LAMP methods have been developed for the detection of the *Hcp100* locus of *H. capsulatum* and for ITS [79,80]. LAMP of the *Hcp100* locus is very specific in laboratory conditions, but less so with clinical specimens [79]. When a positive culture for *H. capsulatum* is used as the gold standard, LAMP of clinical specimens (bone marrow) had low sensitivity and specificity (54% and 64%, respectively) [80]. LAMP has the potential to be useful in a low-resource setting, but further data are needed before use of this technique becomes mainstream.

There is proof of concept that fluorescent in situ hybridization (FISH) targeting *Histoplasma* rRNA may be clinically useful. One study using in-house probes showed that FISH was equally sensitive and specific to in-house PCR and was able to detect *H. capsulatum* in inoculated blood cultures incubated for only 4 h as compared with 10 h with PCR [81]. However, this assay is not currently available for clinical use.

## Data Availability

Data sharing not applicable.
